# A Lentiviral Gene Therapy Strategy for the *In Vitro* Production of Feline Erythropoietin

**DOI:** 10.1371/journal.pone.0045099

**Published:** 2012-09-18

**Authors:** Natalia Vapniarsky, Michael Lame, Samantha McDonnel, Brian Murphy

**Affiliations:** 1 Department of Pathology, Microbiology, Immunology, University of California Davis, Davis, California, United States of America; 2 Department of Molecular Biosciences, University of California Davis, Davis, California, United States of America; 3 Graduate Group of Comparative Pathology, University of California Davis, Davis, California, United States of America; Emory University School of Medicine, United States of America

## Abstract

Nonregenerative anemia due to chronic renal failure is a common problem in domestic cats. Unfortunately, administration of recombinant human erythropoietin often only improves anemia temporarily due to antibody development. In this *in vitro* study, feline erythropoietin cDNA was cloned from feline renal tissue and utilized in the construction of a replication-defective lentiviral vector. The native recombinant feline erythropoietin (rfEPO) sequence was confirmed by sequencing. Upon viral vector infection of human 293H cells, Crandall Renal Feline Kidney cell line and primary feline peripheral blood mononuclear cells, bioactive rfEPO protein was produced. The presence of cellular rfEPO cDNA was confirmed by standard PCR, production of abundant rfEPO mRNA was confirmed by real-time PCR, and secretion of rfEPO protein was demonstrated by Western blot analyses, while rfEPO protein bioactivity was confirmed via an MTT proliferation bioassay. This *in vitro* study demonstrates the feasibility of a replication-defective lentiviral vector delivery system for the *in vitro* production of biologically active feline erythropoietin. Anemic cats with chronic renal failure represent a potential *in vivo* application of a lentiviral gene therapy system.

## Introduction

The glycoprotein hormone erythropoietin, made by the peritubular cells of the renal cortex [1], [2], [3], stimulates the production of red blood cells. Erythropoietin-responsive anemia is a common cause of morbidity in cats affecting 32–65% of cats with chronic renal failure (CRF) [4], [5], [6]. Injection of exogenous recombinant human erythropoietin (rHuEPO) in these cats often results in the resolution of anemia, improvement in appetite, weight gain, energy level, alertness, playfulness, physical strength and attitude [7], [8], [9], [10], [11], [12], [13]. Multiple rHuEPO products are currently available including epoetin alfa (Epogen, Amgen), epoetin beta (Neo-Recormom, Roche) and darbepoetin alfa (Aranesp, Amgen). These products all have the same human-specific primary amino acid sequence but differ in the degree of glycosylation, which affects renal clearance, thus influencing the frequency of administration [14]. Multiple adverse effects have been reported to be associated with the use of rHuEPO in cats, including refractory anemia, systemic hypertension, polycythemia, seizures, vomiting, iron deficiency, injection discomfort, cellulitis, cutaneous/mucocutaneous reactions, and arthralgia [12]. Because the amino acid sequence of rHuEPO is only 81.3% homologous to that of feline EPO, antibodies directed against rHuEPO can develop when it is administered to cats [13], [15], [16]. These antibodies are believed to block rHuEPO bioactivity and potentially cross-neutralize endogenous feline EPO leading to the development of life threatening red cell aplasia (RCA) [17]. Antibodies usually develop within the first few months of rHuEPO administration and a clinically significant immunologic reaction has been reported in 20% to 70% of feline patients receiving rHuEPO [18].

Several therapeutic strategies utilizing species-specific recombinant EPO have been attempted to address the issue of rHuEPO immunogenicity in cats. In an *in vitro* recombinant feline erythropoietin (rfEPO) strategy, Chinese hamster ovary (CHO) cells were transfected with a construct resulting in the production of biologically active, glycosylated rfEPO protein [19]. In a subsequent *in vivo* experiment by a different research group, CHO cell-generated rfEPO was utilized in the treatment of cats with nonregenerative anemia attributed to CRF [17]. In treated cats, the hematocrit increased significantly during the first 3 weeks of treatment. However, some of the cats that initially responded appropriately (8 of 26) developed red cell aplasia (RCA) that was refractory to additional treatments. Importantly, the reported cDNA sequence of the rfEPO utilized in this *in vivo* study had a single nucleotide substitution in the 44th codon (GGG), which resulted in the misincorporation of the glycine amino acid *in lieu* of glutamic acid. In an analysis of the EPO cDNA sequence of multiple mammalian species, the 44^th^ codon of the EPO gene, GAG, encodes the glutamic amino acid, manifesting for the existence of strong evolutionary pressure at this locus [15]. It is conceivable that glycine misincorporation in the rfEPO could lead to immunogenicity in treated cats.

Adeno-associated viral vectors expressing recombinant feline erythropoietin (rAAV-rfEPO) have been developed [2], [16]. A rAAV-rfEPO vector, when administered intramuscularly to normal healthy cats, resulted in an increase in hematocrit over a 7 week period [16]. In a subsequent study, healthy cats treated with the lower-end vector dose had variable to no response while cats treated with the higher dose had significantly increased hematocrits during the first 3 weeks of rfEPO treatment [2]. Antibodies against the recombinant adeno-associated vector were detected in all of the vector treated cats. The authors speculated that this immune response may have prevented or reduced the success of vector readministration. In the same study, one cat developed RCA and another developed pathologic erythrocytosis. The authors speculated that posttranslational differences may exist between the endogenous and rfEPO synthesis intramuscularly [2]. Nevertheless, the genetic code of the rfEPO cDNA utilized in the construction of this viral vector encoded glycine (44_GGG_) in lieu of glutamic acid (44_GAG_) [2].

The ideal gene delivery system for treatment of CRF-associated non-regenerative anemia would have limited or no secondary immunogenicity (*i.e*. lack most or all viral genes), stable and native transgene expression and the ability to transduce both dividing and non-dividing cells. Replication-incompetent lentiviral vectors fulfill this list of requirements [20]. Various lentiviruses have been employed as viral vectors including HIV-1, HIV-2, simian immunodeficiency virus, feline immunodeficiency virus, caprine arthritis encephalitis virus, bovine immunodeficiency virus and visna virus [21]. Lentiviral vector pseudotyping with the vesicular stomatitis virus (VSV) surface glycoprotein confers an ability to transduce a broad range of cell types and increases the vector’s therapeutic applications. In addition, cell-specific tropism can be individually tailored to the desired target cells *via* pseudotyping with the appropriate envelope protein [21]. Previous studies have demonstrated long-term stable gene transfer in experimental models in the absence of immune responses [22], [23], [24], [25], [26]. A lentivirus expressing EPO cDNA has been tested in both normal [27] and uremic rats [28]. In these studies, lentivirus-treated rats had efficient and sustained EPO secretion. To the authors’ knowledge, replication-incompetent lentiviral systems have not been previously evaluated for the production of recombinant rfEPO. In this *in vitro* study, we demonstrate the effective use of a commercially available HIV-1-based lentiviral vector for the production of biologically active rfEPO in feline and human cell lines and in primary feline peripheral blood mononuclear cells.

## Results

### Sequence of feEPO cDNA

**Figure 1:**
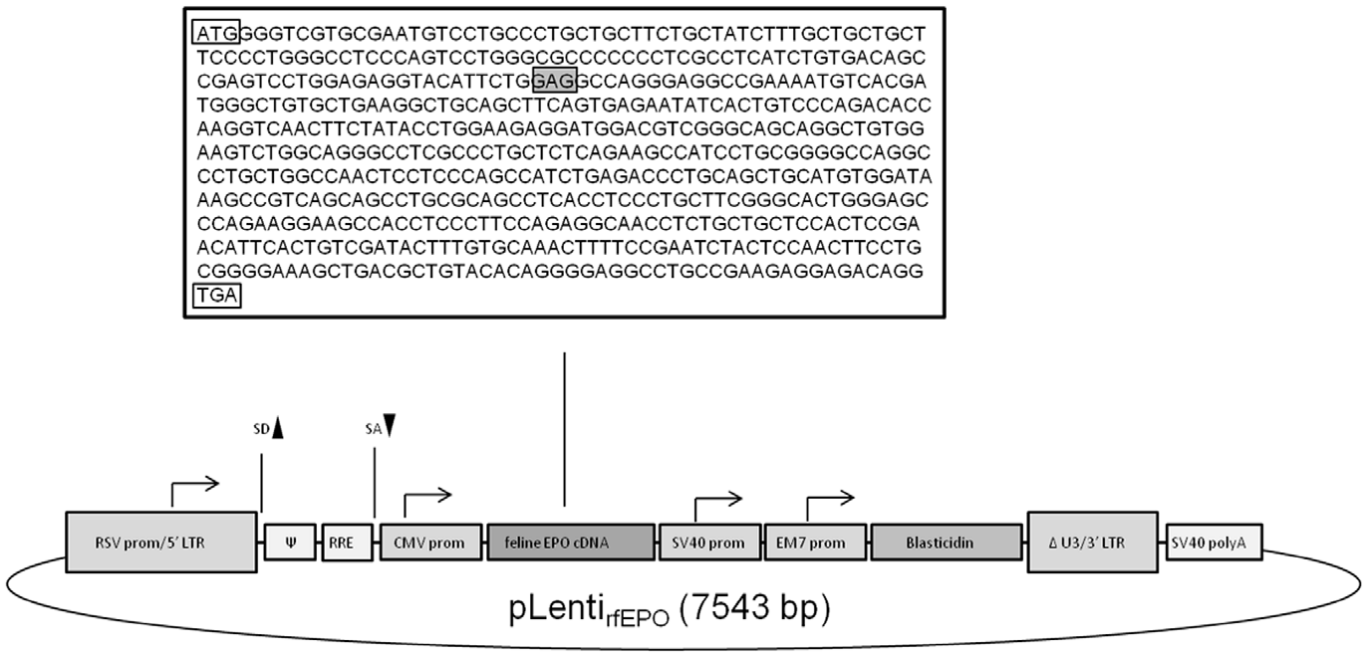
rfEPO sequence and schematic of the pLenti rfEPO vector. The 7543 nucleotide pLenti rfEPO vector, derived from the HIV-1 based pLenti6/V5-D-TOPO (Invitrogen) is depicted schematically along with the cloned fEPO cDNA sequence. For the cDNA sequence, the start (ATG) and stop (TGA) codons are boxed while the 44^th^ codon (GAG) is in a grey box. For the vector schematic, all of the promoter elements are light grey with an accompanying arrow. The 5′ HIV-1 LTR has a 229 nucleotide deletion in U3 replaced with the RSV enhancer/promoter (RSV prom/5′LTR). The 5′ splice donor (SD), splice acceptor (SA), packaging signal (Ψ), Rev response element (RRE), CMV promoter, feline erythopoietin cDNA (579 nucleotides), SV40 early promoter, EM7 promoter, Blasticidin resistance gene and the SV40 polyadenylation signal are depicted schematically. The 3′ HIV-1 LTR has a 52 nucleotide deletion in U3. The pUC origin and Ampicillin (*bla*) resistance gene/promoter are present in the vector backbone but are not depicted.

The feline kidney-derived EPO cDNA was sequenced and is displayed in Figure 1. In this cDNA, the 44^th^ codon codes for glutamic acid (GAG), consistent with the (almost complete) GenBank fEPO sequence L10606. The complete fEPO sequence was submitted to GenBank under the accession number JQ413414.

### Detection of the GFP-EPO Fusion Protein by Fluorescent Microscopy and Western Analysis

**Figure 2:**
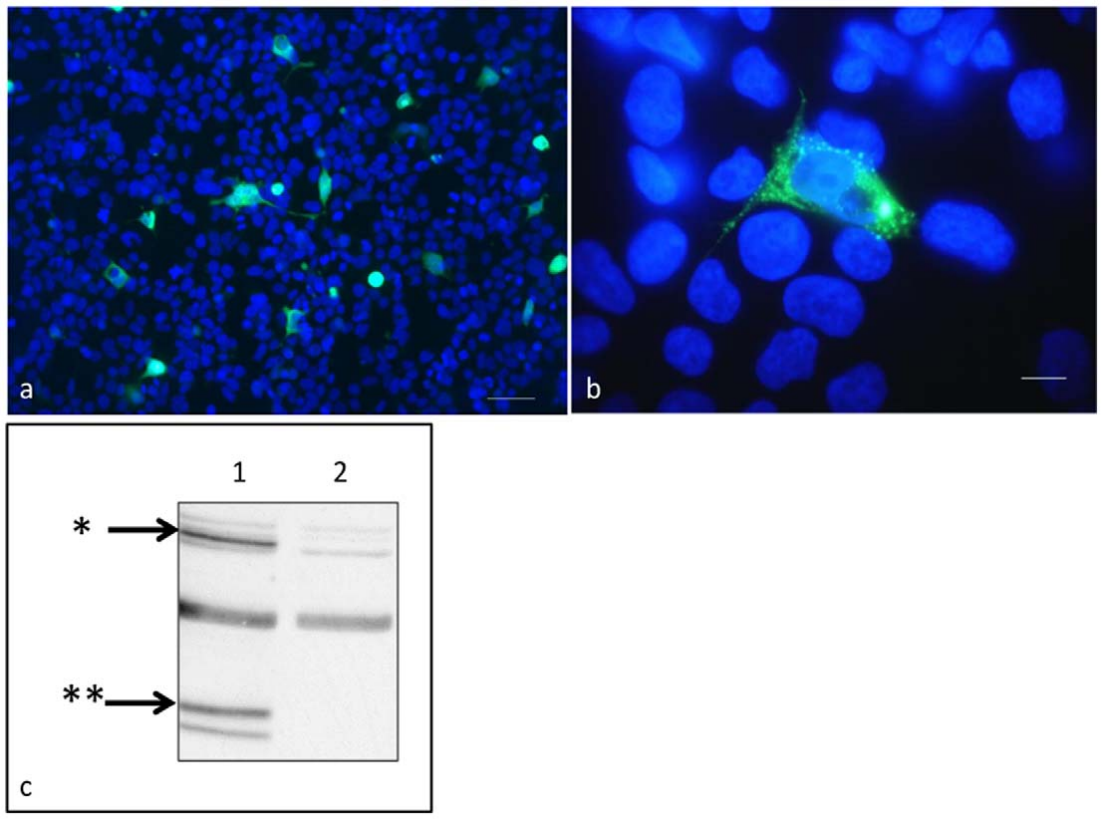
293H Cells transfected with pGFP-EPO demonstrate abundant cytoplasmic fluorescence and GFP antigen expression. a) In a merged image (DAPI/GFP channels), approximately 3% of 293H cells demonstrate positive green fluorescence (DAPI/GFP fluorescence microscopy, 20×magnification, scale bar = 50 µm). b) In a merged image, the transfected 293H cell cytoplasm is filled with numerous fluorescent granules (sequestration of the material into storage granules) (DAPI/GFP fluorescence microscopy, 100×magnification, scale bar = 10 µm). GFP fluorescence is not evident in the nucleus. c) GFP and GFP-EPO fusion protein-specific bands are evident in a Western blot analysis. A dark band is evident in lysates obtained from transfected cells at ∼50 kDa (GFP-EPO fusion protein, *, lane 1) and is not present in lysates from control cells (lane 2). GFP-specific degradation products that are between 25 and 37 kDa are evident in the lane derived from cells transfected with pGFP-EPO (**, lane 1) and not in the lane derived from the control cells (lane 2).

293H cells transfected with pGFP-EPO demonstrate abundant cytoplasmic green fluorescence, consistent with expression of the green fluorescence fusion protein (Figures 2a and 2b). The transfection efficiency was determined to be approximately 3% (Figure 2a). Western analysis confirms the production of a GFP fusion protein (primary anti-GFP antibody, Fig. 2c). GFP (28,973 Daltons) and non-glycosylated feline erythropoietin (20,914) have a combined mass of 49,887 Daltons. A band corresponding to ∼50,000 can be seen in the transfected 293H cells which is absent for control cells. Three distinct GFP-positive degradation products that fall between 25 and 37 kDa are evident in the lane derived from cells transfected with pGFP-EPO and not in the lane derived from the control cells.

### Full Length rfEPO Complimentary DNA Sequence Detected by Standard PCR in Infected PBMC

DNA was isolated from lentiviral vector-infected and uninfected feline PBMC, and standard PCR with primers for the full-length EPO cDNA sequence was performed. Appropriate-sized (579 bp) amplicons were present upon agarose gel electrophoresis only in DNA from cells infected with the lentiviral vector (data not shown).

### Feline EPO Message Detected by Real-time RT-PCR in PBMC Infected with rfEPO Lentiviral Vector

Total RNA was harvested from feline PBMC infected with the lentiviral EPO vector, reverse transcribed into cDNA, and assayed for a 130 bp fragment of the feline EPO sequence *via* real-time PCR. Feline EPO transcripts were detected at a level approximately 100-fold greater than housekeeping GAPDH transcripts (94±3 copies rfEPO cDNA per copy GAPDH cDNA), demonstrating that feline PBMC infected with a replication-incompetent lentiviral vector coding for rfEPO produce abundant feline EPO mRNA. Feline EPO-specific transcripts were below the limit of detection in uninfected control PBMC.

### Feline EPO Protein in 293, CRFK and PBMC Infected with rfEPO Lentiviral Vector

**Figure 3:**
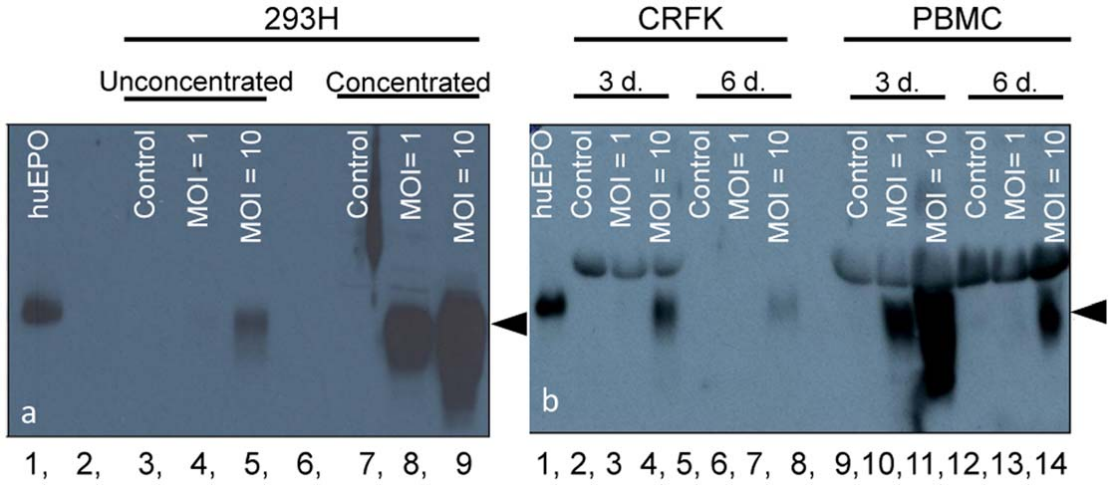
Supernatants of various cells infected with the lentiviral feEPO vector contain abundant FeEPO protein. a) Supernatants collected from 293H cells at 6 days post infection were concentrated using centrifugal filters (lanes 7–9), or used unconcentrated (lanes 3–5). Lanes 3 and 7 represent uninfected control cells, while lanes 4 & 8 and 5 & 9 represent cells infected at an MOI of 1 and 10, respectively. Lane 1 is 10 U of commercial human EPO as a positive control; lanes 2 and 6 are blank. b) Supernatants collected from CRFK (lanes 2–7) and feline PBMC (9–14) at 3 (lanes 2–4, 9–11) and 6 (lanes 5–7, 12–14) days post infection. Lanes 2, 5, 9, & 12 represent uninfected control cells, while lanes 3, 6, 10, & 13 and 4, 7, 11, & 14 represent cells infected at an MOI of 1 and 10, respectively. Lane 1 is 10 U of commercial human EPO as a positive control; lane 8 is blank. Arrowheads: ∼34 kDa determined by molecular weight marker.

Western blot analyses of clarified supernatants derived from 293H (Figure 3a), CRFK and feline PBMC (Figure 3b) infected with the lentiviral feline EPO vector demonstrate appropriate-sized glycoprotein bands (∼34 kDa) for feline erythropoietin, consistent with the human EPO positive control lane. EPO bands were undetectable in protein lysates derived from infected cells (not shown), likely due to the preferential secretion of synthesized rfEPO protein. Supernatants from cells infected at an MOI of 1 and 10 were evaluated 6 days (293H) or 3 and 6 days (CRFK and PBMC) post-infection. Qualitatively, cells infected at an MOI of 10 resulted in greater rfEPO production than in cells infected at an MOI of 1 in all three cell types. In addition, filter-concentrated supernatants from lentiviral-infected 293H cells contained more rfEPO than unconcentrated supernatants (Figure 3a). Finally, the concentration of rfEPO in the supernatant was greater at 3 days than 6 days post-infection in CRFK and PBMC (Figure 3b).

### Detection of rfEPO Bioactivity in Supernatants Derived from 293, CRFK and PBMC Infected with rfEPO Lentiviral Vector

**Figure 4:**
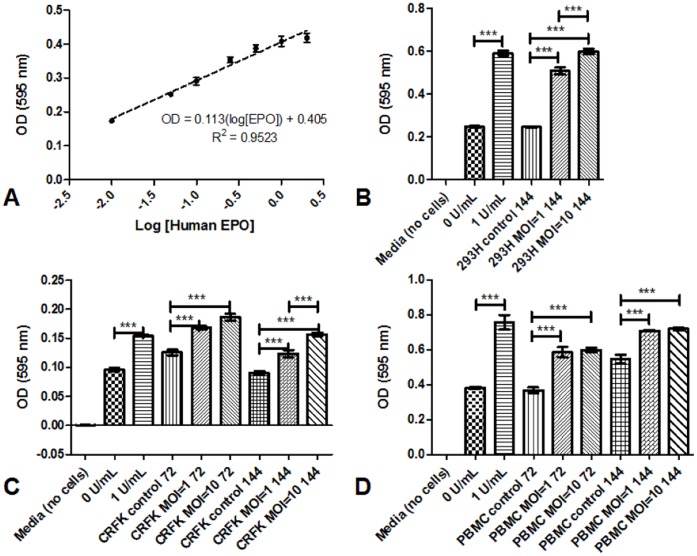
Supernatants of various cells infected with the lentiviral feEPO vector contain biologically active feEPO protein. A) Standard curve generated by serial dilutions of commercial human EPO. MTT bioassay performed using the supernatants from B) 293H, C) CRFK, or D) feline PBMC infected (MOI 1 or 10) with the lentiviral fEPO vector (or uninfected controls) at B) 6 days or (C,D) 3 and 6 post infection. TF-1 cells treated with commercial human EPO (1 U/mL) served as positive controls for the assay, while TF-1 cells incubated in media without EPO (0 U/mL) served as negative controls. Media alone (Media) served as the reference for background absorbance. Error bars represent the standard deviation of triplicate well experiments; asterisks denote significantly different values based on one-way ANOVA with Tukey’s multiple comparison post-test (p<.05).

MTT bioassays (human erythroblastic leukemia cell proliferation) were performed to evaluate the activity of the recombinant rfEPO produced in lentiviral EPO vector-infected cells (Figure 4). A standard curve (r^2^ value of 0.95) was generated by treatment of the human erythroblastic cells with serial dilutions of commercial human EPO from 2 to 0.01 U/mL (Figure 4a). Clarified supernatants from cells infected with the lentiviral EPO vector at an MOI of 1 or 10, and supernatants from uninfected control cells, were likewise used in proliferation assays after 6 days (293H), or 3 and 6 days (CRFK and PBMC) post-infection. Human erythroblastic cells treated with clarified supernatants from lentivirus-infected cells were significantly more proliferative than those treated with supernatants from uninfected controls, demonstrating that the cell supernatants from infected 293H (Figure 4b), CRFK (Fig. 4c), and PBMC (Figure 4d) contain measurable bioactive rfEPO. In general, the bioactivity was greater in cells infected at an MOI of 10 than 1, likely representing a greater concentration of EPO in the supernatant at higher multiplicity of infection.

## Discussion

This study was designed to demonstrate the feasibility of a replication-defective HIV-1 based lentiviral vector for the *in vitro* production of rfEPO in human and feline cell lines as well as in primary feline leukocytes. The results of this study indicate that biologically active rfEPO protein can be produced *in vitro* utilizing this strategy. A long-term potential application for this gene therapy system is the treatment of cats with non-regenerative anemia secondary to CRF.

The concentration of rfEPO was higher in CRFK and PMBC cells after 3 days of incubation relative to samples derived from cells incubated for 6 days, as demonstrated by figure 3. This may be the result of episomally located, non-integrated viral vector activity which gradually diminished with prolonged incubation due to the loss of the episomal DNA. Alternatively, degradation of rfEPO protein may have been the result of supernatant proteases released form disrupted or dying cells.

In two feline erythropoietin cDNA sequences available in the GenBank database, a single nucleotide mismatch is present in the protein coding region of exon 2 at the 44^th^ codon (18^th^ codon in the mature feline EPO, after the signaling peptide is cleaved). One of the GenBank sequences codes for glycine (44_GGG_, accession No. U00685) and the other for glutamic acid (44_GAG_, accession No. L10606) [17]. Studies utilizing the rfEPO sequence coding for glycine have demonstrated several deleterious effects in experimental animals including RCA, and pathological erythrocytosis [2], [17], whereas a study using the rfEPO sequence coding for glutamic acid described no pathology [16]. Whether this mismatch represents focal allelic variation, or alternatively, an artifact introduced during cloning, is controversial. However, in an *in vivo* study utilizing rfEPO protein coding for glycine (44_GGG_), the feline genomic DNA of 12 recipient cats was amplified and sequenced to establish evidence for allelic variation. The nucleotide sequence at this locus in all 12 cats (24 fEPO alleles) was determined to be 44_GAG_, coding for glutamic acid [17]. In a database search of mammalian erythropoietin cDNA sequences (NCBI BLAST), over 50 different species of mammals demonstrate the GAG codon at this site with none coding for GGG. In aggregate, these findings suggest that the “polymorphism” identified at the 44^th^ codon of the feline erythropoietin cDNA most likely represents an artifact introduced during the initial cloning procedures. Importantly, modification of a single amino acid residue in human EPO has been shown to affect the biological function dramatically (two fold reduction in activity) [29].

For *in vivo* applications, the problem of rEPO immunogenicity is not the only important consideration that needs to be addressed. Regulation of EPO transgene dosage may play a critical role in the future success or failure of rfEPO gene therapy attempts. Transgene regulation can be dramatically affected by the function of the promoter driving expression of the rfEPO transgene. Multiple researchers have chosen to use a powerful constitutive promoter such as the cytomegalovirus immediate-early promoter [16]. However, In two different studies, healthy cynomologous macaques treated with macaque-derived EPO-expressing AAV vectors developed supraphysiologic levels of EPO and polycythemia [30], [31]. In an experiment where the recombinant EPO cDNA was expressed constitutively from a cytomegalovirus promoter, severe anemia (RCA) developed in some animals and polycythemia required repeated therapeutic phlebotomies to maintain nontoxic hematocrits [30]. Profound anemia has also been reported in macaques treated with an AAV vector expressing EPO cDNA driven from a doxycycline-regulated promoter [31]. As a result of studies like these, researchers have developed naked DNA-based gene therapy protocols in rodent models [32], [33]. These studies have resulted in correction of anemia without triggering excessive hematopoiesis. Researchers have attempted to regulate the mammalian host hematocrit level by controlling the dosage of the administered recombinant viral vector. Unfortunately, this technique has resulted in an all-or-none phenomenon rather than the clinically desired dose-dependent increase in hematocrit [2]. Excision of the intramuscular rAAV2-rfEPO vector injection site has also proved unreliable for abrogating pathological erythrocytosis of rfEPO [2]. Although the therapeutic window of rfEPO in anemic cats is expected to be large [16], many researchers now feel that clinically useful gene expression of rfEPO should include a mechanism to regulate gene expression to avoid life-threatening anemia or pathological erythrocytosis [2]. An oxygen-regulated gene therapy approach has been successfully employed in erythropoietin-deficient mice [34]. In the same study, mice treated with CMV-EPO constructs acquired fatal polycythemia. The main physiologic stimulus for erythropoietin synthesis is renal hypoxia. When hypoxia is present, hypoxia-inducible factor 1 (HIF-1α) is free to bind to hypoxia–response elements of oxygen regulated genes [14]. These response elements control the erythropoietin gene within the kidneys, and binding of HIF-1α stimulates an increase in production of erythropoietin. A constitutively active CMV promoter was utilized in the *in vitro* lentiviral strategy reported here. As previous *in vivo* animal studies have indicated that the regulation of EPO expression may play a role in hematopoietic pathology [30], [31], [34], a more sophisticated mechanism of transcriptional control may be warranted prior to the establishment of *in vivo* studies.

Insertional mutagenesis induced by viral vectors is an ongoing problem in gene therapy [35]. Vector-induced mutagenesis has been a concern ever since 4 of 9 human patients treated for X-linked severe combined immunodeficiency developed leukemia following treatment with a retroviral vector [36]. As a result of this, lentiviral vectors with increased safety properties were developed. Nevertheless, proliferative hematopoietic disorders have recently been described in mice [37] and human patients treated with modern lentiviral vectors [36]. This has resulted in the design of ever more sophisticated lentiviral vector safety features including the incorporation of suicide genes, cell or tissue specific promoters, local/regional delivery of viral vectors, locus-targeted transgene integration and the use of “insulators” to prevent oncogene activation [36].

To provide an additional measure of control, the replication-defective lentiviral vector system creates the potential for influencing cell-specific tropism *via* vector pseudotyping. Selection of certain viral envelope glycoproteins or other proteins facilitates cell targeting to enhance directed gene transfer [21]. For example, cell-specific targeting has been achieved through the use of lentiviral vectors pseudotyped with the Rabies virus glycoprotein [38] or the CD4 receptor [39]. A variety of envelope-like genes are currently available to provide vector-target cell specificity [21]. Optimally, vector targeting of long-lived cells could potentially abrogate the need to continuously readminister the viral vector. An optimal lentiviral vector may include hypoxia response element-regulated expression of rfEPO cDNA and the incorporation of a suicide gene into the vector design. A combination of these various strategies may provide a clinically relevant *in vivo* method for the treatment of non-regenerative anemia associated with CRF in cats.

## Materials and Methods

### Extraction and Cloning of Feline Erythropoietin

Normal feline renal tissue was harvested within 20 minutes of euthanasia from a feline cadaver. The renal tissue was obtained from a cat that was humanely euthanized for reasons unrelated to this experiment, and submitted for the disposal and group cremation at Veterinary Medical Teaching Hospital Pathology at UC Davis. The owners permitted unrestricted use of the cat’s organs. The cat was not euthanized for the purposes of this study and the renal tissue was obtained one time only. The humane euthanasia procedure was performed according to the best standards of care. Based on our institution regulations, no IACUC protocol is required for the use of unrestricted organs obtained post mortem. The renal tissue was rinsed with ice cold diethylpyrocarbonate-treated isotonic phosphate buffered saline and approximately 1 gram of renal tissue was subsequently reduced to a fine powder with liquid nitrogen cooled mortar and pestle. Aliquots of frozen renal tissue were dissolved in Trizol reagent (Invitrogen) and total RNA was isolated according to the manufacturer’s specifications with the exception that the RNA-containing aqueous phase was mixed 1∶1 with 70% ethanol and passed over an RNeasy mini column (Qiagen) including an on column DNase (Qiagen) digestion step. Messenger RNA was isolated from total RNA using an Oligotex mRNA kit (Qiagen) and was converted into cDNA using SuperScript III (Invitrogen) and random hexamers.

Feline erythropoietin cDNA was amplified using gene-specific primers based upon NCBI accession number NM_001009269.1 (forward 5′- ATG-GGG-TCG-TGC-GAA-TGT-CCT-GC; reverse ‘5-TCA-CCT-GTC-TCC-TCT-TCG-GCA-GGC-CTC). The reaction was catalyzed with Expand High Fidelity^Plus^ blended Taq and proof reading protein (Roche). Reaction conditions were as follows: an initial 94°C dissociation stage for 2.5 min followed by 10 cycles at 94°C for×30 s, 59°C 30 s, and 72°C for 45 s and incremented 10 s/cycle thereafter for an additional 25 cycles, followed by a 10 min final elongation at 72°C. The resulting amplicon was electrophoresed and the 579 bp band corresponding to the predicted cDNA molecular weight was extracted using a QIAquick Gel extraction kit (Qiagen). The fresh PCR amplicon was inserted into a green fluorescence protein (GFP) fusion vector (pcDNA3.1/NT-GFP-TOPO, Invitrogen) according to the manufacturer’s instructions generating pGFP-EPO. Resulting plasmids were cloned and expanded in TOP10 chemically competent *E. coli* (Invitrogen) and sequenced by a local vender (Davis Sequencing).

### Cells

Human fetal kidney (293H) cells were purchased from GIBCO (Invitrogen), 293FT (Invitrogen), HT1080 (Stratagene) and Crandell Rees feline kidney (CRFK) cells were purchased from ATCC. Whole blood was collected in 5 mL vacutainer tubes containing EDTA from specific pathogen-free (SPF) cats (Feline Research Laboratory, Center for Companion Animal Health, UC Davis), and peripheral blood mononuclear cells (PBMC) were harvested by density gradient centrifugation through Ficoll-Hypaque (Sigma).

### Erythropoietin-GFP Fusion Protein Transfection and Visualization

Fluorescent microscopy was utilized to determine if cells transfected with pGFP-EPO vector appropriately expressed the GFP fusion protein. 293 H cells grown in 75 cm^2^ flasks were trypsinized and seeded into 60 mm diameter culture dishes (Nunclon) and allowed to progress to 80% confluence. Cells were grown in DMEM (Gibco) containing 20% fetal bovine serum (FBS, Hyclone), 1X non-essential amino acids (Gibco), penicillin (100 U/mL) and streptomycin (100 µg/mL) antibiotics (HyClone). Cells were transfected with pGFP-EPO according to the manufacturer’s instructions (Lipofectamine 2000, Invitrogen). Seventy-two hr post-transfection, the cells were dislodged with trypsin and transferred to glass cover slips pre-coated with human recombinant fibronectin (18.75 µg/22 mm^2^). Cells were allowed to attach overnight, the coverslips were washed with PBS and then fixed for 30 minutes in 2.5% paraformaldehyde in PBS. The fixative was changed with 0.1% Triton-X-100 in PBS containing 1 µg/ml of DAPI (Invitrogen), the reaction was allowed to progress for 5 min, followed by 3×5 min washes with 0.1% Triton-X-100 in PBS. Coverslips were mounted with Prolong Gold antifade reagent (Molecular Probes). Fluorescent microscopy was performed using an Olympus BX61 microscope coupled to a Penguin 600CL/150CL digital camera system (Pixera Corporation). Photography was completed within 96 hours post-transfection.

### Lentiviral Vector Construction

The feEPO cDNA was transferred from pGFP-EPO without the GFP cDNA, to the lentiviral expression vector pLenti6/V5 (Invitrogen) using PCR technology. The following primers were employed, forward 5′-CACC-ATG-GGG-TCG-TGC-GAA-TGT-3′, reverse ‘5-TCA-CCT-GTC-TCC-TCT-TCG-GCA-G-3′. The reverse primer codes for a STOP signal (TGA) at the 3′ end of EPO coding sequence. PCR conditions were the same as the cloning reaction with the exception that the annealing temperature was dropped to 54°C. After the initial amplification, the resulting amplicon was blunted with Pfu (Stratagene), agarose gel purified as previously specified, and inserted into the pLenti6/V5 directional TOPO vector (Invitrogen) as specified by the manufacturer. The resulting plasmid, containing the rfEPO cDNA but lacking the GFP gene (named pLenti_rfEPO_, Figure 1) was propagated and selected in Stbl3 *E. coli* (Invitrogen). Potential rearrangement in the LTR regions was checked by digesting the plasmid with Afl II and XhoI restriction enzymes. The plasmids were sequenced by a local vender (Davis Sequencing).

### Virus Production

Lentivirus-infected 293 FT cells were used to generate pseudotyped viral particles. 293FT cells were grown to 70% confluence in 10 cm diameter plates using DMEM (Gibco) containing 10% FBS (Hyclone), 1x MEM non-essential amino acids (Gibco), 500 µg Geneticin/ml, and penicillin (100 U/mL) and streptomycin (100 µg/mL) antibiotics (HyClone). Cells were transfected with Lipofectamine 2000 (Invitrogen) according to manufacturer’s instructions with 3 µg of pLenti_rfEPO_ plus a 1∶1:1 molar mixture of packaging plasmids pLP1, pLP2, and pLP/VSVG (Invitrogen). Media containing pseudotyped lentivirus was harvested 51 hr post transfection, passed through a Millex HV 0.45 µm filter (Millipore), and concentrated using Amicon Ultra centrifugal filters 50 kDa cut off (Millipore). Lentivirus was stored at -80°C until used.

Lentivirus was titered using HT1080 cells as recommended by Invitrogen. Virus was added at 1/100, 1/1000, and 1/10,000 dilution in volumes of 50 µL; a control was included which was devoid of virus. After 24 hours, media was replaced with 4.5 ml DMEM and again 24 hours later with the addition of a selecting agent, 10 µg Blasticidin/ml. Selection progressed until all cells in the control flask were dead and then colonies were counted in the flasks receiving virus. The total yield for 2×10 cm plates was 6×10^7^ viral particles.

### Nucleic Acid Detection and Quantification in Feline PBMC

SPF feline PBMC were grown in RPMI media (Gibco) with 10% FBS (Hyclone), 100 U recombinant human IL2/ml (Hoffman-La Roche), and containing penicillin (100 U/mL) and streptomycin (100 µg/mL) antibiotics (HyClone). Cells were plated in 12-well tissue culture dishes and infected with the lentiviral EPO vector at an MOI of 10. Uninfected PBMC propagated in parallel were used as a negative control. Two days post infection, cells were harvested by centrifugation and DNA was isolated *via* QIAamp DNA mini kit (Qiagen) while RNA was isolated using TRIzol reagent (Invitrogen) according to the manufacturers’ protocols. Isolated RNA was DNase treated (TURBO DNase, Ambion) according to the manufacturer’s protocol, and further purified using a commercial RNA clean-up protocol (RNeasy kit, Qiagen). Approximately 1 µg RNA was reverse-transcribed into cDNA (First Strand cDNA Synthesis System, Origene); a control sample was run without reverse transcriptase.

Standard PCR was performed on DNA samples with the forward and reverse primers used for lentiviral vector construction (above), and amplicons were electrophoresed on agarose gel post-PCR to visualize bands corresponding to the full-length EPO cDNA sequence. Real time PCR assays were performed on cDNA samples in triplicate with Real Mastermix SYBR Rox (5 Prime) on a 7300 Real Time PCR System (Applied Biosystems), and subsequently analyzed with the 7300 system software. Both infected and uninfected control PBMC cDNA were assayed for a 130 bp amplicon at the 3′ terminus of the EPO coding sequence (forward primer 5′-AACCTCTGCTGCTCCACTCC-3′, reverse primer 5′-TCACCTGTCTCCTCTTCGGCAG-3′), as well as feline housekeeping gene product GAPDH (forward primer 5′-AAATTCCACGGCACAGTCAAG-3′, reverse primer 5′-TGATGGGCTTTCCATTGATGA-3′) in parallel. Negative controls (no template and reverse transcriptase negative samples) were run simultaneously to ensure there was no nucleic acid contamination. EPO and GAPDH Ct values were converted to copy number using standard curves generated by serial dilution of plasmid DNA containing the cloned EPO or GAPDH sequences.

### Western Blot

Human 293H and feline CRFK cells were grown in DMEM media (HyClone) with 10% fetal bovine serum (FBS, Gemini Bioproducts), and feline PBMC were grown in RPMI media (HyClone) with 10% FBS and 100 U/mL recombinant human IL2 (Hoffman-La Roche); both cultures contained penicillin (100 U/mL) and streptomycin (100 µg/mL) antibiotics (HyClone). Cells were plated in 25 cm^2^ cell culture flasks (293H, CRFK) or 12-well tissue culture dishes (PBMC), and were infected with the lentiviral EPO vector at a multiplicity of infection (MOI) of both 1 and 10. Uninfected cells were used as controls.

Clarified supernatants were harvested at 6 days (293H) or 3 and 6 days (CRFK and PBMC) post-infection, mixed 1∶1 with 2x loading buffer (Hoefer Scientific Instruments), boiled for 2 min, and loaded onto 10–20% SDS-PAGE gels (Expedeon). Aliquots of each 293H cell supernatant were concentrated using 3 kDa centrifugal filters (Milipore) prior to use. Five microliters (10 U) of commercially available human EPO (Epoetin alpha, EPOGEN) were used as a positive control, and protein size was determined with a molecular weight marker (Expedeon). Samples were electrophoresed and electrotransfered to a PVDF membrane (Biorad) according to the gel system manufacturer’s instructions (PAGEgel). The PVDF membrane was rinsed twice in tris-buffered saline with 0.1% Tween-20 (TBS/T) wash buffer, blocked for 1 hr with 5% nonfat milk/TBS/T (blocking buffer), and washed three more times before being incubated overnight in 1∶1000 polyclonal rabbit anti-human EPO antibody (Accurate Chemical & Scientific Corporation) in blocking buffer at 4°C (as above). The membrane was washed three times, and incubated for 1 hr in a 1∶10,000 dilution of goat anti-rabbit peroxidase conjugated antibody (Thermo Scientific) in blocking buffer, washed three more times, and incubated in ECL reagent (Pierce) for 1 min. Autoradiographs were created by exposing ECL-treated membranes to Scientific Imaging Film (Kodak) for 1–10 min, and developed with a medical film processor (Konica Minolta Medical & Graphic).

Western analyses for the GFP-erythropoietin fusion protein were conducted as follows. Cells were cultured and transfected as described previously. Cells in 60 mm diameter dishes were washed with ice cold PBS 2x and then dislodged by scraping with Costar Cell lifters (Corning Incorporated). Cells were sedimented at 400×g for 3 minutes and then disrupted with 150 mM NaCl, 50 mM Tris (pH, 7.4), 1% NP40, 0.25% Sodium Deoxycholate, 0.1% SDS, 1x Protease Inhibitor cocktail Set I (Calbiochem), 1 mM NaF, and 1 mM activated Na_3_VO_4_(RIPA). The reaction was allowed to progress for 1 hour on ice and the cell debris was pelleted at 16,000×g for 30 minutes. The protein concentration was determined for both naïve 293H (control) and transfected cell supernatants, and adjusted with RIPA to the same protein concentration per volume. Protein lysates were mixed with 4×Laemmli sample buffer and boiled for 3 min. Lysates were applied at 75 µg per lane and separated on an 11% T 2.75% C SDS-PAGE gel (140×120.5×1.5 mm) with a 4% stacking gel. Proteins were transferred to PVDF overnight at 10°C. The membrane was blocked for 1 hr with 5% blotting grade non-fat milk (BioRad) in TBS (blocking buffer) and subsequently incubated overnight at 4°C with mouse anti-GFP antibody (UC Davis/NINDS/NIMH NeuroMab Facility) diluted 1∶1000 in blocking buffer. The blot was subsequently washed 3×with TBS and then incubated for 1 hr with sheep HRP-anti-mouse antibody (GE Healthcare, 1/5000) diluted in blocking buffer. The blot was washed 1×with 0.05% Tween-20 in TBS followed with 3×with TBS. Film detection was as previously described except ECL Plus Western Blotting Detection System was employed (GE Health Care) coupled with Hyperfilm (Amersham).

### MTT Bioassay

To demonstrate bioactivity of recombinant feline erythropoietin, 293H, CRFK, and feline PBMC were grown in OPTIMEM (GIBCO) media with penicillin/streptomycin antibiotics as above, and infected with the lentiviral EPO vector at an MOI of 1 and 10 in the presence of polybrene (5 µg/ml, Sigma). Supernatants were harvested at 6 days (293H) or 3 and 6 days (CRFK and PBMC) post-infection; uninfected cell supernatants were used as a control. Human TF-1 erythroblastic cells (ATCC), were plated in a 96-well tissue culture dish at 5×10^4^ cells/well in RPMI media with 5% FBS and penicillin/streptomycin antibiotics (as above), and incubated with clarified supernatants for 48 hrs. TF-1 cells+1 U/mL human EPO (Epoetin alpha, EPOGEN) were used as a positive control, and TF-1 cells+fresh media, or fresh media alone, were used as negative controls. After the 48 hour incubation, an MTT cell proliferation assay (ATCC bioproducts) was performed per the manufacturer’s protocol, and the optical density of the wells was determined at 595 nm using a Thermomax microplate reader (Molecular Devices). The MTT assay was performed in triplicate for each sample, supernatant or control. One-way ANOVA with Tukey’s multiple comparison post-test was performed using GraphPad Prism version 5.04 for Windows (GraphPad Software, San Diego, CA). A standard curve was created using serial dilutions of human EPO from 0.01 U/mL to 2 U/mL, and the MTT assay was performed as described previously.

### Statistics

Quantifiable MTT bioassay data was performed in triplicate, means and standard deviations were determined (GraphPad). For an experimental set of data, a global one-way ANOVA test was performed. Where significant global differences were detected, Tukey’s multiple comparison tests were made. Differences were determined to be significant for p values less than 0.05.
